# Radial extracorporeal shock wave therapy for the management of spasticity in cerebral palsy: study protocol for a randomized controlled trial

**DOI:** 10.3389/fneur.2024.1402452

**Published:** 2024-06-13

**Authors:** Míriam Tur Segura, Francisca Gimeno Esteve, Tamara Biedermann Villagra, Jordi Jiménez Redondo, Nicolás García Rodríguez, Raimon Milà Villarroel

**Affiliations:** ^1^Fundació Aspace Catalunya, Barcelona, Spain; ^2^School of Health Sciences Blanquerna, Ramon Llull University, Barcelona, Spain

**Keywords:** shock wave therapy, radial extracorporeal shock wave therapy, rESWT, spasticity, cerebral palsy

## Abstract

**Background:**

Spasticity is the most common motor disorder in cerebral palsy (CP), and its management is complex, posing a significant challenge for the rehabilitation team. Radial extracorporeal shock wave therapy (rESWT) has emerged in recent years as an effective, non-invasive, and low-risk alternative for the management of spasticity in CP patients, with only minor side effects such as small bruises or discomfort during application. There is great variability in rESWT administration protocols, ranging from a single session up to the 12 sessions. The most extensively studied protocol involves 3 rESWT sessions with a one-week interval between session. According to current literature, the effect of rESWT has not been investigated by extending the time interval between sessions beyond 1 week to determine if therapeutic effects on spasticity can be prolonged over time.

**Methods:**

Following a power calculation using the minimal clinical important difference of our primary outcome (R2 of Modified Tardieu Scale), 72 patients will be included in the study. Enrolment is based upon inclusion/exclusion criteria outlined in the Methods section. Participants will be randomized in 3 groups. Each patient will receive 2000 impulses in the Triceps Sural muscle (distributed by all the plantar flexor muscles: soleus and gastrocnemius), at a 2.2 Bars pressure and a frequency of 8 Hz. The Control Group will receive 3 rESWT sessions with a time interval of 1 week between each session. The Experimental Group A will receive 3 rESTW sessions with a time interval of 2 weeks between each session and the Experimental Group B will receive 3 rESTW sessions with a time interval of 4 weeks between each session.

**Discussion:**

This study will provide further information regarding the effect of rESWT on spasticity in patients with CP. If an increase in the time interval between rESWT sessions allows for the prolongation of therapeutic benefits on spasticity, it will be clinically relevant fact. With the same treatment dosage, patients will be able to benefit from its effects for a longer period of time.

**Clinical trial registration:**

ClinicalTrials.gov, identifier NCT05702606.

## 1 Introduction

Cerebral palsy (CP) is a neurological developmental condition that begins in early childhood and persists throughout life. It is defined as “a group of permanent disorders of movement and posture development, causing limitations in activity and attributed to non-progressive disturbances that occur in the fetal or infant brain development. Motor disorders in cerebral palsy are often accompanied by alterations in sensation, perception, cognition, communication, and behavior, as well as epilepsy and secondary musculoskeletal problems” (1). According to the data provided by the latest systematic review with meta-analysis available on the worldwide prevalence of CP, which includes forty-one regions across 27 countries on five continents, it is estimated that the prevalence of CP in high-income countries is 1.6 per 1,000 live births, while in low- and middle-income countries, it is 3.4 per 1,000 live births. CP remains the leading cause of childhood disability (2).

Spasticity is the most common motor disorder in CP (3). It is one of the signs of upper motor neuron syndrome and is characterized by an increase in stretch reflexes that varies depending on the speed of the stretching (4). Spasticity is a component of the multifaceted motor disability of CP, and although it may not be the primary factor interfering with function, participation, or activity (5), it can play an important role in limiting patients ability to mobilize and engage in daily activities by interfering with limb function, causing pain secondary to muscle spasms, which can disturb sleep (6, 7) and in the long term, lead fixed contractures and musculoskeletal deformities (8).

The management of spasticity in CP is complex and presents a great challenge for the rehabilitation team. There is considerable variation in the management of spasticity, including the availability of treatments (physical therapy, pharmacological interventions, orthopaedic interventions, surgical options, etc.) and the intensity of their use (9).

The objectives of his therapeutic approach include; reduce pain, facilitate the use of orthopaedic aids, improve posture, minimize contractures and deformity, facilitate mobility and dexterity, with the ultimate goal of maximizing the patient’s potential and promoting their independence and quality of life (10).

Several studies have demonstrated the favorable effect of extracorporeal shock wave therapy (ESWT) in the treatment of spasticity in patients with cerebral palsy, multiple sclerosis, traumatic brain injury, and stroke (11–14). Although the mechanism of action of ESWT remains unknown, current evidence suggests that it induces the production of nitric oxide, which modulates neurotransmission at neuromuscular junctions; triggers a cascade of biological responses, including the expression of angiogenesis-related growth factors, leading to an antifibrotic effect through increased blood flow and enhanced tissue regeneration; induces degeneration of acetylcholine receptors; and may selectively target the terminal plaques of neuromuscular junctions (15). Two types of extracorporeal shock waves are used in medical therapy; radial extracorporeal shock waves (rESW) and focused extracorporeal shock waves (fESW). Both are single acoustic impulses with an initial positive peak pressure between approximately 11 Megapascals (MPa) rESW and more than 100 MPa fESW, reached in less than 1 μs. There are no comparative studies on which of the two types of shockwave therapy, rESW or fESW, is more effective for addressing spasticity in patients with CP. However, there are comparative studies between these two therapies in the management of spasticity in stroke patients, where the results indicate that rESW may have a slightly better effect on the spasticity, and studies on rESW are of higher methodological quality and level of evidence (13).

Radial extracorporeal shock wave therapy (rESTW) has emerged in recent years as an effective, non-invasive, non-pharmacological and low-risk alternative for the management of spasticity in CP patients, with only minor side effects such skin redness or discomfort during application. Current literature agrees that rESWT treatment is effective in improving spasticity in individuals with CP; this effect has been studied primarily in the Triceps Surae or plantar flexor muscle. It has been shown that the effect of shock waves is maintained up to 12 weeks after treatment. Positive effects have been observed in the reduction of spasticity, leading to improvements in the Modified Tardieu Scale (MTS), in the Modified Asworth Scale (MAS), in the passive range of motion (pROM), in increasing of the Gross Motor Function Measurement (GMFM) and plantar support surfaces measured with force platforms, among others outcomes. Since rESWT is a relatively new approach for the management of spasticity, there is significant variability in administration protocols, particularly regarding the number of sessions (ranging from a single session to 12 sessions) and the time interval between sessions. The most extensively studied protocol involves 3 rESWT sessions with a one-week interval between each session. According to current literature, no study has conducted a follow-up longer than 12 weeks, and the effect of rESWT with extended time intervals between sessions beyond 1 week has not been investigated to determine whether the therapeutic effects on spasticity can be prolonged over time (16–24).

The aim of this study is to assess whether increasing the time interval between rESWT sessions extends the therapeutic benefits on spasticity to be prolonged in patients with CP.

## 2 Methods and analysis (including design; selection/treatment of subjects; interventional methods; data analysis)

### 2.1 Study design

**Figure 1:**
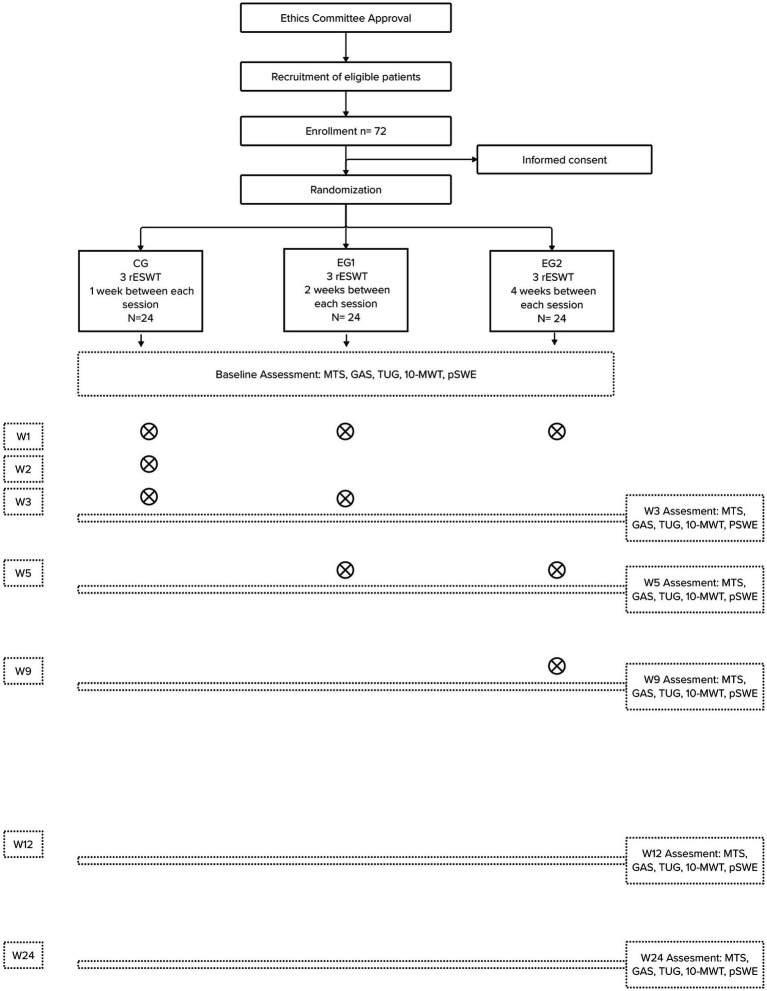
The flow diagram for this study. rESWT, radial Extracorporeal Shock Wave Therapy; MTS, Modified Tardieu Scale; GAS, Goal Attainment Scaling; TUG, Timed Up and Go Test; 10-MWT, 10 Meter Walk Test; pSWE, Point Shear Wave Elastography; X, Session of rESWT; W, Number Week.

This clinical trial is designed as a single-center randomized clinical trial with three parallel groups. This protocol follows the Consolidated Standards of Reporting Trials (CONSORT) Statement on randomized trials and it will be conducted according to the Recommendations for Interventional Trials (SPIRIT). A flowchart overview of the study is presented in Figure 1. The Standard Protocol Items: SPIRIT table for enrolment, interventions, and assessments is presented in Table 1.

**Table 1:** The standard protocol items: SPIRIT table for enrolment, interventions, and assessments.

	**Enrolment**	**Allocation**	**Intervention**					**Follow-up**	
Timepoint	-24 week	0	1 week	2 week	3 week	5 week	9 week	12 week	24 week
Enrolment									
Eligibility screen	x								
Informed consent	x								
Allocation		x							
Interventions									
CG			x	x	x				
EGA			x		x	x			
EGB			x			x	x		
Assessments									
MTS			x		x	x	x	x	x
GAS			x		x	x	x	x	x
TUG			x		x	x	x	x	x
10MWT			x		x	x	x	x	x
pSWE			x		x	x	x	X	x

Tabulation of enrolment, interventions, and assessments throughout the trial. CG – Control Group (3 rESWT sessions 1 week between each session). EGA - Experimental Group A (3 rESWT sessions 2 week between each session). EGB – Experimental Group B (3 rESWT sessions 4 week between each session). MTS – Modified Tardieu Scale. GAS - Goal Attainment Scaling. TUG - Timed Up and Go Test. 10MWT - 10 Meter Walk Test. pSWE - Point Shear Wave Elastography.

Seventy-two participants with cerebral palsy will be recruited. Patients will be randomized into 3 groups. All patients will be followed for 24 weeks. The trial was approved by the local medical and ethical commission (reference number PR-2021-16) and is registered in ClinicalTrials.gov (NCT05702606).

### 2.2 Objectives

The aim of this study is to assess whether increasing the time interval between rESWT sessions extends the therapeutic benefits on spasticity to be prolonged in patients with CP. The specific objectives are: (a) to evaluate whether the effects of the time factor imply an improvement in spasticity assessed with the Modified Tardieu Scale, (b) to evaluate whether the effects of the time factor imply an improvement in the clinical and functioning aspects of the person, assessed with the Goal Attainment Scaling, (c) to evaluate whether the effects of the time factor imply an improvement in the functional response of improving the gait and the overall patient mobility, (d) to evaluate whether the effects of the time factor imply a decrease in muscle contracture produced by spasticity and assessed with point shear wave elastography using musculoskeletal ultrasound, (e) analyze whether the patient’s age influences the treatment results, (f) analyze whether the level of gross motor function classification influences treatment results, (g) know the patient’s satisfaction regarding the different interventions with shock waves, (h) record possible adverse effects of interventions.

### 2.3 Setting study

The study will be conducted at Fundació Aspace Catalunya, a non-profit organization founded in 1961. The objectives of this entity have always been to promote services for the research, diagnosis, rehabilitation, and education of people with CP or other developmental disabilities, as well as their families. It was a pioneer in Spain in the treatment of patients with brain damage, mainly congenital, and especially in CP.

### 2.4 Participants

The patients eligible to participate in the study will be, in all cases, from the foundation and will be selected by the medical rehabilitators of the organization. Once it is confirmed that participants meet the selection criteria (Table 2), they will be provided with a letter that informs them about the purpose, objectives, necessity of the study, its duration, procedures, and potential benefits and risks. Those patients who express their willingness to participate will be given the informed consent form to accept the study conditions. In the case of minor patients, even if the informed consent is signed by their legal representatives, it must be signed in their presence and with their consent. Patients and/or legal representatives will also be informed of their rights and the option to withdraw from the study at any time without providing reasons. A withdrawal/abandonment form will be provided to them.

**Table 2:** In-and exclusion criteria.

Inclusion criteria	Exclusion criteria
Patients with a CP classified as spastic	Having received shockwave therapy treatment in the Triceps Sural or any other muscle of the lower extremity within the 6 months prior to the study
Patients of both sexes	
Age between 4 and 45 years	Having received botulinum toxin treatment and/or focal intramuscular treatment with phenol or alcohol in the Triceps Sural or any other muscle of the lower extremity within the 6 months prior to the study
Spasticity in the Triceps Surae muscle	
Who present a Gross Motor Classification (GMFCS) level l and ll	Any contra-indication for the specific treatment (e.g., coagulopathies, malignancies in treated area)
With unilateral or bilateral involvement	Patients who have undergone surgical intervention for orthopaedic foot deformities in the past year
Having obtained the informed consent signed by the participant or their legal guardian	Fixed deformities in the ankle joint
	Clinical signs of myopathy and neuropathy

### 2.5 Randomization, blinding and treatment allocation

Once the participants and/or their legal representatives have signed the informed consent, they will be randomized through an equally probable algorithm using the statistical program R software to determine which of the 3 interventions they will be assigned to. Because the age range for the inclusion of participants is wide, it will be minimized with a stratification of the sample into three age ranges. From 4 to 15 years, from 16 to 30 years and over 30 years. In this study, the evaluating physiotherapist and their assistant will be blinded throughout the entire intervention. The data analyst will also be blinded. Due to the nature of the technique employed in the study, neither the patient nor the professional administering the rESWT sessions can be blinded. The concealment of the assignment will be centralized through one of the researchers, who will be responsible for communicating the assigned intervention to the participants and/or their families via a telephone call. The assignment outcome will be recorded in the corresponding section of the data collection notebook, which will not be accessible to the evaluating physiotherapist. This section of the data collection notebook will be integrated into the complete patient dossier once the follow-up for the last patient is completed, ensuring that the evaluator does not have access to this information during the course of the study. The evaluating physiotherapist will always be the same and will be unaware of the origin of the intervention group to which the patient belongs. Neither the evaluating physiotherapist nor their assistant will inquire about the treatment received, nor will they ask any questions to the doctors, physiotherapists in the center, or patients regarding the intervention groups. Instructions will also be given to the patient and their family not to discuss the received treatment during assessments. Interventions and evaluations will take place in different locations within the center.

### 2.6 Interventions

Each patient will receive 2000 impulses in the Triceps Sural muscle (distributed by all the plantar flexor muscles: soleus and gastrocnemius), at a 2.2 Bars pressure and a frequency of 8 Hz. For the application of the therapy the patient will be positioned lying face down on a stretcher. Conductive gel will be applied, and a sweeping motion will be performed across the entire area to be treated. The application will have a duration of 4 min for each muscle group. In patients with bilateral involvement, treatment will be administered to both extremities. The application of rESTW does not require analgesia, sedation or anesthesia. No adverse effects are expected from the intervention beyond those observed in only two studies, such as temporary skin redness, petechiae, or superficial small hematomas that resolve within a few days after application (16, 21).

The Control Group will receive 3 rESWT sessions with a time interval of 1 week between each session.

The Experimental Group A will receive 3 rESTW sessions with a time interval of 2 weeks between each session.

The Experimental Group B will receive 3 rESTW sessions with a time interval of 4 weeks between each session.

For treatment in all groups, the same device and probe will always be used: the Swiss Dolor Clast Smart model, which guarantees cavitation at any frequency, from the company EMS (Electro Medical System) based in Nyon, Switzerland. The equipment complies with all European safety regulations required for medical devices. Registration: EN-60601-1, Class I. Type BF IP40. 93/42 EEC.

During the course of the study and the follow-up period, patients will not be able to receive botulinum toxin treatment and/or focal intramuscular treatment with phenol or alcohol in the Triceps Sural or any other muscle of the lower extremity. All patients will continue to undergo their regular physiotherapy sessions or physical activity. The type of exercises performed and the total hours per week will be recorded.

### 2.7 Outcome measures

Primary outcome measures: Spasticity of the treated muscles assessed with the Modified Tardieu Scale (MTS). Secondary outcome measures: Goal Attainment Scale (GAS), Timed Up and Go Test (TUG), 10 Meter Walk Test (10-MWT), Point shear wave elastography (pSWE), satisfaction with the therapy, and adverse effects. Prior to the first rESWT session, the blinded evaluating physiotherapist and his assistant will conduct a baseline assessment (t0) of all outcome variables and other aspects deemed appropriate as reference for assessing goal achievement. These assessments will be repeated across all intervention groups at 3 weeks from the start of treatment (t1), 5 weeks from the start of treatment (t2), 9 weeks from the start of treatment (t3), 12 weeks from the start of treatment (t4), and 24 weeks from the start of treatment (t5).

### 2.8 Clinical assessments

#### 2.8.1 Modified Tardieu Scale

The main variable of the study will be the physiological response of spasticity in the treated muscle, assessed using the Modified Tardieu Scale (MTS). This test is based on the evaluation of muscular resistance to passive stretching at two different speeds: slow speed (V1) and fast speed (V3), to quantify the joint angles at which such resistance appears. The V1 stretch is used to determine the angle of muscle resistance at low speed (R2), equivalent to the passive range of motion (pROM) and the V3 stretch is used to determine muscle resistance at high speed (R1) or catch reflex. The MTS it could be more sensitive than other measures in assessing spasticity. Its inter- and intra-rater reproducibility is acceptable (25). All measurements were conducted using a inclinometer to determine the angular value of the two segments. For the measurement of calf muscles, separate stretches were applied to the gastrocnemius muscle (measured with the knee extended) and the soleus muscle (measured with the knee flexed at 90°).

#### 2.8.2 Goal attainment scaling

The Goal Attainment Scaling (GAS) is an individualized and patient-centered scale in which specific goals (one main and one secondary in our case) are proposed with concrete expectations for each patient. It provides two types of information: quantitative (assessment of success) and qualitative (what the patient aims to achieve). Once the objectives of shock wave application are agreed upon with the patient and/or family, such as improving function, gait, pain, perceived spasticity sensation, or enhancing orthosis tolerance, improvement expectations will be determined for each objective. It will be assessed whether these expectations have been met (0), are better (+1), much better (+2), worse (−1), or much worse (−2) than expected. It is a highly dynamic scale that allows quantifying any set objective. It is possibly the scale that best aligns clinical practice with patient expectations (26).

#### 2.8.3 Timed up and go test

The functional response in improving the overall patient mobility will be assessed using the Timed Up and Go Test (TUG), where the time it took for the person to stand up from a chair, walk 3 meters, return, and sit back down with their back against the chair (27). The time will be measured in seconds with a stopwatch. The patients will undergo the test twice with the instruction to “walk as fast as they can without running.” The best recorded time will be documented.

#### 2.8.4 10 Meter Walk Test

The functional response in improving gait will be evaluated with the 10 Meter Walk Test (10MWT), where the time it took for the person to walk 10 meters in a straight line will recorded (28). The patients will undergo the test twice with the instruction to “walk as fast as they can without running.” The best recorded time will be documented.

#### 2.8.5 Point shear wave elastography

Elastography by ultrasound is a non-invasive imaging technique that measures tissue displacement, i.e., tension in response to the application of a specific force. The Point Shear Wave Elastography (pSWE) measures the acoustic force impulse transmitted perpendicular to the tissue, providing a value in kPa based on the greater or lesser deformity of the explored tissue (29). The muscular stiffness of the soleus and gastrocnemius muscles will be assessed. The Aplio a550 device from Canon Medical Systems SA will be used to perform the measurements.

#### 2.8.6 Satisfaction with the therapy

A satisfaction questionnaire will be administered to assess the level of satisfaction with the treatment among all patients and/or legal guardians.

### 2.9 Adverse effects

If adverse effects arise from any of the interventions, they will be systematically recorded and documented in the data collection notebook specifically created for the study.

### 2.10 Data management

All data will be recorded and stored in the Data Collection Notebook (DCN). These will be kept in a locked cabinet by the person responsible for clinical assessments. All data will be recorded in an ACCESS-type database with input coding. The first section: Will collect all the baseline personal information of the patient: age, sex, type of CP, and description according to the guidelines of the European CP Registry using the GMFCS. The second section: Will collect all the information related to baseline assessments and temporal assessments of each variable. In this part of the notebook, only the evaluating physiotherapist will have access. The third section: Will collect all the information regarding any adverse effects that may appear during the study.

### 2.11 Sample size estimation

The sample size has been calculated using the GRANMO program, accepting an alpha risk of 0.05 and a beta risk of 0.2. It requires 24 subjects for each group, totaling 72 patients, to detect a difference equal to or greater than 5 degrees of improvement in R1 and R3 of MTS. It is assumed that the common standard deviation is 16.44, with a correlation coefficient between the initial and final measurement of 0.91. These data have been extracted from a study on the treatment of spasticity using shockwave therapy in patients with CP (21). A follow-up loss rate of 10% will be estimated.

### 2.12 Statistical analyses

Statistical analysis of the data will be conducted using SPSS software version 23 and R-software (MLMM package). An intention-to-treat analysis will be performed. Significance level 5% (*p*-value <0.05). For the descriptive analysis, a normality check will be conducted using Shapiro–Wilk / Kolmogorov–Smirnov tests. For quantitative variables, the mean and standard deviation will be reported (in case of non-parametric data, the median and interquartile range). For qualitative variables, frequency and percentages will be reported. For inferential statistics, a mixed linear model for repeated measures will be used and adjusted as necessary for covariates such as age, sex, GMFCS, and other comorbidities that may be observed. For the qualitative analysis, semi-structured interviews will be conducted, and the following categories will be established: Physical efficacy, bodily function (subcategories: increased relaxation, mobility, balance, etc.). Autonomy, daily life activities, and functionality (frequency, intensity, location). Pain conditions (intensity, location). Rating of satisfaction with shockwave therapy. Subjective, emotional, and related aspects.

## 3 Discussion

Spasticity is the most prevalent motor disorder in cerebral palsy, and its management is intricate, posing a significant challenge for the rehabilitation team. While it may not be the primary factor hindering patient function, participation, or activity (5), it can play a crucial role in restricting their ability to move and perform daily life activities by interfering with limb function, leading to secondary pain from muscle spasms that may even disrupt sleep (6, 7). ESWT is a relatively new therapy in the field of neurology. In 2010, the first clinical trial applying focal shock waves for managing spasticity in patients with cerebral palsy was published (30). Over the recent years, it has established itself as an effective, non-invasive alternative with minimal side effects for spasticity management (11, 31, 32). Currently, there are limited published studies on ESWT for spasticity management in patients with cerebral palsy, and only 9 studies have investigated the effects of rESWT (16–24). The most studied muscle group has been the Triceps Surae, and there is significant disparity in the treatment doses applied in each study, especially regarding the number of sessions and the time interval between sessions. One of the most commonly used protocol is 3 sessions of rESWT with a one-week interval between each session (16, 21). Several features distinguish this study from previous ones. Despite the variability in dose administration, none of them have investigated the effect of rESWT by extending the time interval between sessions beyond 1 week to determine if therapeutic effects on spasticity can be prolonged by applying the same dose. Published studies have not followed up with patients beyond 12 weeks. In this study, we propose a follow-up of patients up to 24 weeks. Current literature indicates that a single session of shockwave therapy showed positive effects up to 4 weeks (17), and 3 sessions with a one-week interval between each session up to 12 weeks (16, 21, 22). Since our study hypothesis is that extending the interval between shockwaves sessions beyond 1 week may prolong the therapeutic benefit on spasticity, we deem it appropriate to extend the follow-up period to 24 weeks to observe potential differences between the study groups. In this study the physiological response of shock waves on the spastic muscle will be evaluated using MTS and pSWE, simultaneously, the functional response in overall mobility and gait of patients will be assessed using TUG and 10MWT. Furthermore, this is the first study to utilize GAS to define therapeutic goals that each patient wishes to achieve individually with rESWT application and analyze the success of therapy according to their specific improvement expectations for each set goal. If the study hypothesis is proven and an increased time interval between shock wave sessions extends therapeutic benefits on spasticity, it would be clinically significant, with the same treatment dose, patients could potentially benefit from shock wave effects for a longer duration. Additionally, this could provide patients and/or their caregivers with a broader range of scheduling options for treatment visits, for instance, it could improve accessibility for individuals who face geographical constraints or challenges in balancing work and family life. In such cases, the ability to attend treatment sessions every two or 4 weeks, instead of three consecutive weeks, could be more advantageous. The results of this study could have widespread implications for the cerebral palsy community, as they may be applicable to similar centers or institutions. Research on the effects of shock waves in reducing spasticity in patients with cerebral palsy opens up a promising avenue for future studies in this field.

## 4 Ethics and dissemination

The study follow the ethical principles for medical research involving human subjects of the Declaration of Helsinki, adopted by the 18th General Assembly of the World Medical Association (World Medical Association, 1964), which were last revised at the association’s 64th General Assembly, in Fortaleza, Brazil, in October 2013. The study protocol has been approved by the medical and ethical commission FIDMAG Germanes Hospitalàries (reference number PR-2021-16) and is registered in ClinicalTrials.gov (NCT05702606). The patients/participants and/or legal representatives provided their written informed consent to participate in this study. The personal data of the research study will be processed in accordance with the General Data Protection Regulation (EU) 2016/679 and with the Organic Law on Data Protection and Digital Rights Guarantees (Law 3/2018, of December 5). The legal basis for the processing will be the explicit, transparent, and unequivocal consent of the data subject, and the purposes of the processing and all the information required by the GDPR Regulation will be informed in all cases. In addition, the measure of pseudonymization will be applied to the processing of data to provide additional security.

## Ethics statement

The studies involving humans were approved by FIDMAG Germanes Hospitalàries (reference number PR-2021-16). The studies were conducted in accordance with the local legislation and institutional requirements. Written informed consent for participation in this study was provided by the participants' legal guardians/next of kin.

## Author contributions

MT: Writing – original draft, Writing – review & editing. FG: Writing – original draft, Writing – review & editing. TB: Writing – review & editing. JJ: Writing – original draft, Writing – review & editing. NG: Writing – review & editing. RM: Supervision, Writing – original draft, Writing – review & editing.
